# Comparing the Efficacy of an Identical, Tailored Smoking Cessation Intervention Delivered by Mobile Text Messaging Versus Email: Randomized Controlled Trial

**DOI:** 10.2196/12137

**Published:** 2019-09-27

**Authors:** Inger Torhild Gram, Dillys Larbi, Silje Camilla Wangberg

**Affiliations:** 1 Norwegian Centre for E-health Research University Hospital of North Norway Tromsø Norway; 2 Department of Community Medicine Faculty of Health Sciences UiT The Arctic University of Norway Tromsø Norway; 3 Department of Health and Caring Sciences Faculty of Health Sciences UiT The Arctic University of Norway Tromsø Norway

**Keywords:** eHealth, electronic mail, mHealth, mobile phones, randomized controlled trial, smoking cessation, text-messaging

## Abstract

**Background:**

There is a need to deliver smoking cessation support at a population level, both in developed and developing countries. Studies on internet-based and mobile phone–based smoking cessation interventions have shown that these methods can be as effective as other methods of support, and they can have a wider reach at a lower cost.

**Objective:**

This randomized controlled trial (RCT) aimed to compare, on a population level, the efficacy of an identical, tailored smoking cessation intervention delivered by mobile text messaging versus email.

**Methods:**

We conducted a nationwide 2-arm, double-blinded, fully automated RCT, close to a real-world setting, in Norway. We did not offer incentives to increase participation and adherence or to decrease loss to follow-up. We recruited users of the website, slutta.no, an open, free, multi-component Norwegian internet-based smoking cessation program, from May 2010 until October 2012. Enrolled smokers were considered as having completed a time point regardless of their response status if it was 1, 3, 6, or 12 months post cessation. We assessed 7315 participants using the following inclusion criteria: knowledge of the Norwegian language, age 16 years or older, ownership of a Norwegian cell phone, having an email account, current cigarette smoker, willingness to set a cessation date within 14 days (mandatory), and completion of a baseline questionnaire for tailoring algorithms. Altogether, 6137 participants were eligible for the study and 4378 participants (71.33%) provided informed consent to participate in the smoking cessation trial. We calculated the response rates for participants at the completed 1, 3, 6, and 12 months post cessation. For each arm, we conducted an intention-to-treat (ITT) analysis for each completed time point. The main outcome was 7-day self-reported point prevalence abstinence (PPA) at the completed 6 months post cessation. We calculated effect size of the 7-day self-reported PPA in the text message arm compared with the email arm as odds ratios (ORs) with 95% CIs for the 4 time points post cessation.

**Results:**

At 6 months follow-up, 21.06% (384/1823) of participants in the text message arm and 18.62% (333/1788) in the email arm responded (*P*=.07) to the surveys. In the ITT analysis, 11.46% (209/1823) of participants in the text message arm compared with 10.96% (196/1788) in the email arm (OR 1.05, 95% CI 0.86-1.30) reported to have achieved 7 days PPA.

**Conclusions:**

This nationwide, double-blinded, large, fully automated RCT found that 1 in 9 enrolled smokers reported 7-day PPA in both arms, 6 months post cessation. Our study found that identical smoking cessation interventions delivered by mobile text messaging and email may be equally successful at a population level.

**Trial Registration:**

ClinicalTrials.gov NCT01103427; https://clinicaltrials.gov/ct2/show/NCT01103427

## Introduction

### Background

Tobacco use is, and has been for many years, one of the leading preventable causes of disease and death. The number of diseases that are established to be smoking related continues to increase [1,2]. Tobacco consumption is decreasing in the developed countries but increasing in the developing countries [3,4]. Although a high proportion of smokers will try to quit, only 2% to 3% will be successful each year [5].

There is a need to deliver smoking cessation support at a population level, both in developed and developing countries.

Studies on internet-based and mobile phone–based smoking cessation interventions have shown that these methods can be as effective as other methods of support, and they can have a wider reach at a lower cost [6-14]. The randomized controlled trials (RCTs) included in the most recent Cochrane reviews on internet-based [13] and mobile phone–based [12] smoking cessation interventions most frequently compare the effect of the intervention with the comparing condition at 6 months post cessation.

Several of these RCTs had different incentives to increase participation and decrease the loss to follow-up. This could be multiple follow-ups using the internet, email, or mobile phone if users did not respond [14-17], by payment for mobile phone use [18,19], by free Nicotine Replacement Therapy [15,20], by gift certificates [14], and by internet-based counseling from nurses [21] or tobacco treatment specialists [22]. However, as pointed out by Eysenbach, electronic health (eHealth) research studies with a high dropout or high loss to follow-up should not be looked upon as failures but rather a natural and typical feature of eHealth interventions that should be expected [23].

In a previous smoking cessation intervention RCT, we compared tailored with nontailored cessation support delivered by email. At 12 months follow-up, 11.2% of the 419 participants who had received the tailored email reported smoking cessation with similar results in the nontailored email arm [24]. This inspired us to design another RCT, at a population level, that would be fully automated, close to a real-world setting, and have high privacy protection. We decided to follow the recommendations from the Society for Research on Nicotine and Tobacco subcommittee on biochemical verification; that large-scale population studies are one of few settings for which biochemical verification is not required and may not be desirable [25]. We wanted to compare the efficacy of tailored smoking cessation support delivered by 2 modalities: text messages and emails.

Both email and mobile phone text message deliveries are easy to set up. The 2 methods have different strengths and weaknesses. Emails are easily deployable, inexpensive, and can deliver long, complex messages. Text messages may have some special advantages for delivering health behavioral interventions compared with emails. Mobile phones are now considered essential, everyday items and are owned by most adults. The *always-with-you* nature of the mobile phone and the intrusiveness or *push* factor of text messages makes this a simple, low-commitment way to receive smoking cessation support. One disadvantage with text messages is that there may be a fee. Another disadvantage may be that they are limited to 160 characters of text. However, in a previous study about diabetes education, the participants reported that they perceived the text messages as urgent and that the shorter format made the messages easier to understand and remember [26]. As we were not sure whether a short message was an advantage or a disadvantage, we decided to use the same tailored messages in the 2 arms.

### Objective

This RCT aimed to compare, on a population level, the efficacy of an identical, tailored smoking cessation intervention delivered by mobile text messaging versus email. We hypothesized that smokers who were allocated to the text message arm compared with the email arm would be more or equally successful at achieving 7-day self-reported point prevalence abstinence (PPA) at the completed 6 months post cessation.

## Methods

### Trial Design

We conducted a nationwide 2-arm, double-blinded, fully automated RCT, close to a *real-world setting*, in Norway. We did not offer incentives such as free medication, other gifts, or personal counseling to increase the participation and adherence rate and to decrease the loss to follow-up. We did not request biochemical verification of smoking cessation.

### Recruitment

We recruited from smokers using an open, free, multi-component Norwegian internet-based smoking cessation program, from May 2010 until October 2012. This website was a part of the Norwegian Directorate of Health’s program to promote smoking cessation. The Directorate promoted the website through newspapers, internet, radio, and television (public service announcements) 3 times during the trial period. Enrolled smokers were counted as having completed a time point regardless of their response status if it was 1, 3, 6, or 12 months post cessation. The Regional Committee for Medical Research Ethics approved the study.

### Participants

At the start of enrollment, an estimated 94% of the adult population had access to the internet in their homes and 96% owned a mobile phone [27]. We assessed 7315 participants using the following inclusion criteria: knowledge of the Norwegian language, aged 16 years or older, ownership of a Norwegian cell phone, having an email account, current cigarette smoker, willingness to set a cessation date within 14 days (mandatory), and completion of a baseline questionnaire for tailoring algorithms. Altogether 4378 of the 6137 participants (71.33%) who were eligible for the study provided informed consent for the smoking cessation trial.

### Randomization and Blinding

A Web-based online random number generator [28] automatically assigned the participants to a text message arm or email arm. The study was double-blinded at enrollment, so neither the participants nor researchers knew in which arm the participants were enrolled. We do not know if any of the participants discovered during the trial that the purpose of the study was to compare the efficacy of tailored messages delivered by text versus email or about their allocation.

We excluded 53 participants (29 consent withdrawn and 24 missing information and double allocation). The remaining 4335 participants took part in the RCT with 2188 (50.47%) participants in the text message arm.

### Implementation

We used Drupal version 6 [29], an open source content management system, to create an automated system that performed all the study procedures (informed consent, registration, randomization, baseline and follow-up questionnaires, and intervention messages). To protect privacy, the data management system consisted of 2 dispatcher servers (A and B).

Dispatcher A sent emails containing a hyperlink to a baseline questionnaire on day 1 and follow-up questionnaires at completed 1, 3, 6, and 12 months post cessation. If there was no response to the questionnaires after 7 days, Dispatcher A sent one reminder email with the same hyperlink.

Dispatcher B applied an algorithm to the responses from the baseline questionnaire and created tailored smoking cessation advice that was delivered either by text message or email. We have described the tailoring algorithm in detail elsewhere [16]. Table 1 shows examples of messages related to personalization and cessation date from the intervention. 

**Table 1:** Examples from the intervention.

Type of message and time of delivery		Question	Answer	Responses
**Personalization**				
	At enrollment	What would you like us to call you?	Jane	—^a^
	180 days after cessation date	—	—	Congratulations, Jane. Today you have been smoke free for a half year!
**Cessation date**				
	At enrollment	When do you intend to stop smoking?	Exact date	—
	5 days after cessation date	—	—	There is no longer nicotine present in your body
**Step down**				
	10 days before cessation	Would you like to do a step-down of your smoking?	Yes	Create a smoke free zone in your home
**Descriptive**				
	2 days before cessation date	Are you currently working?	Yes, working full time	Consider which situations at work that is tempting you to smoke
**Social pressure**				
	58 days after cessation	Do your friends smoke?	Yes, all of them	Watch out! Some of them might like it if you fail. It could make them feel better

^a^Not applicable.

Dispatcher B created a maximum of 150 individually tailored messages. It delivered the first message 14 days before and the last message 12 months after the stated cessation date. Dispatcher B sent daily messages in the beginning, then the frequency decreased gradually during the first 3 months with a substantial fall in frequency after that. More than half of the messages had been sent to the participants 3 months after the cessation date.

The participants in both arms could read the tailored advice directly without logging on to the website. All users had a personal profile on the website showing their progress, that is, days abstained from smoking, money saved, number of cigarettes not smoked, days since last cigarette, today’s advice, cessation calendar with previous advice, and an overview of the social network features. The users could participate in social networking with discussion forums, post questions and advice, and read questions and answers from other users. The users could invite friends (smokers and nonsmokers) to post encouraging messages to them.

### Baseline Registration and Data Collection

The baseline questionnaire asked about sex, age, education in years (0-9, 10-12, 13-16, >16), occupational status (8 categories), number of previous cessation attempts, motivation to cessation (4-point scale), and nicotine dependence as measured by the Fagerstrom Test for Nicotine Dependence (6 items, 10-point scale) [30]. It was optional to answer the descriptive background questions. We had less than 5% missing data for the different questions, except age. A technical error caused the system to not record the age variable correctly at enrollment. We re-introduced this variable as a mandatory question in the baseline questionnaire. Age as an inclusion criterion was not disturbed by this technical error. For each user, the program automatically gathered the total number of log-ins to the website, use of the forum (yes, no), posting new topics (yes, no), replies (yes, no), diary entries (yes, no), and number of entries in another person’s guestbook.

### Outcomes

We calculated response rates and 7-day self-reported PPA for enrolled smokers at completed 1, 3, 6, and 12 months post cessation. The main outcome was the 7-day self-reported PPA at 6 months post cessation. PPA is an assessment of cessation status at a particular point in time when these questions are asked. It is independent of previous answers from the participants. We used the following 2 questions: *Are you currently smoking?* and *Have you been smoking, even as little as one single puff during the past 7 days?* Those who answered “No” to both questions had achieved 7-day self-reported PPA at that specific time point.

### Sample Size

A total number of 540 participants were needed per arm at 12 months post cessation to detect a difference of 5% for 7-day PPA (ie, 15% vs 10%) based on a significance level of 5% and 80% power. We did an interim analysis, almost 2 years into the study. The results showed that the enrollment of smokers had been much slower, and the difference between the 2 arms was smaller than anticipated. We therefore extended the enrollment period by 6 months until October 2012. We also changed the time point for the main outcome to 6 instead of 12 months post cessation so we would have a larger sample size and more power to detect a real difference between the 2 arms.

### Statistical Methods

We recruited smokers continuously, so the number of enrolled smokers in the study and the number of participants who had completed each time point varied throughout the study period. For each arm, we calculated the response rate for the 4 (1, 3, 6, and 12 months) post cessation time points, as the number of participants who had responded to the email questionnaire at that time point divided by all enrolled smokers who had completed that time point. For each arm, we conducted an intention-to-treat (ITT) analysis and calculated the 7-day self-reported PPA for the completed time points. We calculated the number of participants who reported to have achieved 7-day PPA divided by all enrolled smokers who had completed that time point. This means that all nonresponders, who had completed a time point, were counted as smokers. We used chi-square test statistics to compare, by arm, the selected characteristics at baseline and the time point–specific response rates. We calculated effect size of the 7-day self-reported PPA in the text message arm compared with the email arm as odds ratios (ORs) with 95% CIs for the 4 time points post cessation. A 2-sided *P* value of <.05 was considered statistically significant. All analyses were conducted using IBM SPSS Statistics version 21.

## Results

### Study Population

Baseline data were available for 4335 (50.5% text message arm) smokers. At enrollment, the median age was 39 years for both arms. In the text message arm (n=334), the age range was from 16 to 72 years, and in the email arm (n=338), it was from 16 to 71 years.

Table 2 shows that more than 70% of the participants were females, more than 60% reported to have at least 13 years of education, and the majority was employed full time. The table shows that the distribution of the selected characteristics at baseline did not vary according to study arm (all *P* values >.13), confirming that the randomization process had worked as it should.

The use of the website’s guestbook, diary, and forum and number of log-ins were low and did not differ between the 2 arms (data not shown).

### Consolidated Standards of Reporting Trials’ Diagram

**Figure 1:**
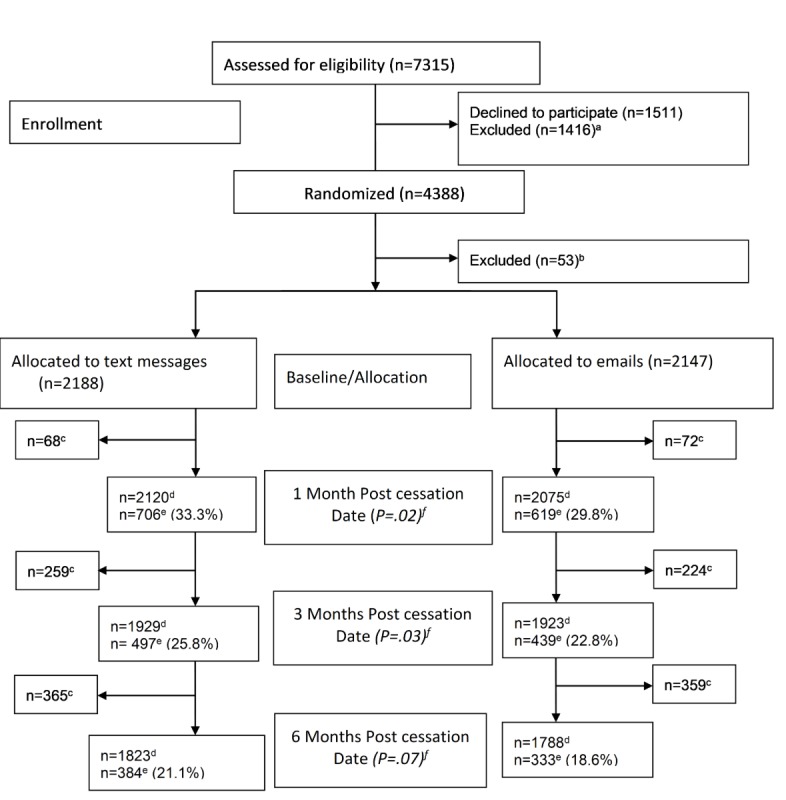
Consolidated Standards of Reporting Trials’ diagram. Randomized controlled trial, Norway 2010-2012 (N= 4335). a – already stopped smoking (n=631); did not complete baseline registration (n=517); not smoking cigarettes (n=20); referred to substudy (n=248). b – text message/email arm; consent withdrawn n=29 (17/12); missing/double allocation n=24 (12/12). c – participants that had not completed the next follow-up time point. d – Participants included in the analysis. e – responders to follow-up email questionnaire. f – Chi-square statistics; P value for difference between the 2 arms.

Figure 1 shows that the response rates were higher in the text message arm compared with the email arm at 1 and 3 months (both *P* values <.05) but not at 6 months (*P*=.07; Figure 1). At 12 months post cessation, the response rate was 25.3% (238/941) in the text message arm and 22.7% (210/927) in the email arm for participants who had completed that time point (*P*=.18).

**Table 2:** Distribution of selected characteristics at baseline (N=4335) by study arm.

Selected characteristics^a^		Total, n (%)	Text message arm, n (%)	Email arm, n (%)	*P* value^b^
**Sex (N=4335)**			2188 (50.47)	2147 (49.53)	.41
	Male	1214 (28.00)	626 (28.00)	588 (27.38)	
	Female	3121 (71.99)	1562 (72.00)	1559 (72.61)	
**Education (years; N=4322)^c^**			2180 (50.44)	2142 (49.56)	.56
	0-9	305 (7.1^d^)	161 (7.38)	144 (6.7)	
	0-12	1446 (33.46)	722 (33.11)	724 (33.8)	
	13-16	1707 (39.50)	848 (38.89)	859 (40.1)	
	>16	864 (20.0)	449 (20.59)	415 (19.4)	
**Occupation (N=4334)**			2188 (50.49)	2146 (49.95)	.70
	Employed, full-time	2423 (55.91^d^)	1221 (55.91)	1202 (56.01)	
	Employed, part-time	543 (12.52)	263 (12.02)	280 (13.0)	
	Retired	32 (0.73)	13 (<1)	19 (<1)	
	Home keeper	69 (1.59)	35 (2)	34 (2)	
	Student	490 (11.30)	265 (12.1)	225 (10.5)	
	Disability	266 (6.1)	133 (6.1)	133 (6.2)	
	Rehabilitation	267 (6.2)	138 (6.3)	129 (6.0)	
	Unemployed	244 (5.6)	120 (5.5)	124 (5.8)	
**Cessation attempts (N=4335)**			2188 (50.47)	2147 (49.53)	.47
	Never	651 (15.0)	324 (14.80)	327 (15.2)	
	Once	728 (16.8)	370 (16.91)	358 (16.7)	
	Twice	992 (22.9)	505 (23.08)	487 (22.7)	
	3 times	646 (14.9)	309 (14.1)	337 (15.7)	
	>3 times	1318 (30.40)	680 (31.1)	638 (29.7)	
**Motivation score (N=4319)^c^**			2181(50.50)	2138(49.50)	.14
	1 (very weak)	74 (2)	46 (2)	28 (1)	
	2 (pretty weak)	483 (11.2)	235 (10.8)	248 (11.6)	
	3 (pretty strong)	2691 (62.31)	1374 (63.00)	1317 (61.60)	
	4 (very strong)	1071 (24.80)	526 (24.1)	545 (25.5)	
**Fagerstrom Test for Nicotine Dependence score (N=4237)^c^**			2138 (50.47)	2098 (49.53)	.72
	0-3 Low	1245 (29.39)	622 (29.1)	623 (29.7)	
	4-6 Medium	2725 (64.33)	1386 (64.83)	1339 (63.82)	
	7-10 High	266 (6.3)	130 (6.1)	136 (6.5)	

^a^Given as numbers (%).

^b^Chi-square statistics; *P* value for difference between the 2 arms according to sex, education, occupation, motivation score and Fagerstrom Test for Nicotine Dependence score.

^c^Some numbers vary owing to missing values.

^d^Some percentages add up to more than 100 owing to rounding.

### Smoking Cessation

Table 3 shows that 11.46% (209/1823) in the text message arm compared with 10.96% (196/1788) in the email arm reported 7-day PPA at 6 months post cessation (OR 1.05, 95% CI 0.86-1.30). A similar ITT analysis for the 12-month post cessation time point revealed that the 7-day self-reported PPA was 12.2% (115/941) in the text message arm and 13.6% (126/927) in the email arm (OR .89, 95% CI 0.68-1.16).

**Table 3:** Seven-day self-reported point prevalence abstinence (PPA), among enrolled smokers who had completed the 1-, 3-, and 6-month post cessation time point by arm and the corresponding likelihood odds ratio (95% CI) comparing the text message arm with the email arm.

Completed time point post cessation	7-day self-reported PPA (%) in text message arm divided by total enrolled^a^ (N=2188), n (%)	7-day self-reported PPA (%) in email arm divided by total enrolled^a^ (N=2147), n (%)	Email arm reference at the corresponding time point	Likelihood odds ratio (OR) with 95% CI) In text message arm compared with email arm
1 month	2120 (19.1)	2075 (19.0)	1.00	1.01 (0.86-1.18)
3 months	1929 (14.6)	1923 (14.6)	1.00	1.00 (0.84-1.20)
6 months	1823 (11.5)	1788 (11.0)	1.00	1.05 (0.86-1.30)

^a^Intention-to-treat analyses.

## Discussion

### Principal Findings

The main result from this large, nationwide, double-blinded RCT was that for those who had completed the time point at 6 months post cessation, the identical program delivered by text messages and emails was equally effective at supporting smoking cessation. In both arms, 1 in 9 enrolled smokers had achieved 7-day self-reported PPA at 6 months post cessation. Similarly, the response rate to the program was 1 in 5 enrolled smokers in both arms at this time point. Furthermore, this RCT conducted at a population level, close to a real-world setting, found that smokers may successfully achieve 7-day PPA at 6 months post cessation without having received incentives such as free medication, other gifts, or personal counseling. Another finding was that a smoking cessation intervention RCT on a population level scale can be fully automated. The program also had a long-term component of 12 months which very few smoking intervention RCTs have.

We find it promising that the tailored interventions delivered by text messages were equally successful as those delivered by email at both 6 and 12 months post cessation.

### Comparison With Past Work

To our knowledge, only the UK txt2stop RCT [19], with close to 3000 participants in the intervention arm that received smoking cessation text messages, is larger than our comparable text message arm. In the UK trial, at 6 months post cessation, the ITT analyses revealed that the smoking cessation rate was doubled in the intervention arm (9%) compared with the control arm (4%). The latter received text messages unrelated to quitting [19]. This RCT used continuous smoking abstinence that had biochemical verification.

The previously referred Cochrane review on mobile phone interventions included a total of 12 RCTs. The RCTs varied in how they measured the smoking abstinence outcome from how the UK trial provided the 6 months post cessation outcome to how we measured it. The overall result from the Cochrane meta-analysis showed that 1 in 11 (9%) smokers with support from text messages and 1 in 18 smokers with no program support managed to be abstinent at 6 months post cessation. In total, 9 of the 12 RCT studies enrolled less than 500 persons in each arm and all the studies stopped at 6 months post cessation [9].

In another recent review on mobile phone interventions, 17 of the 20 studies (85%) included had follow-up that was shorter than 6 months post cessation [11].

Our RCT had, in each arm, more than 300 responders at 6 months and more than 200 responders at the 12-month post cessation time point, with close to 1000 participants in each arm that had completed the 12-month post cessation time point. We find it motivating that neither the loss to follow-up nor the achieved 7-day self-reported PPA declined from the completed 6- to 12-month post cessation time point in our trial.

Muench et al have discussed the beneficial features of mobile phone text messages as a tool for smoking interventions. They find that text messages are perceived as more of a personal form of communication and are more likely to be read quickly, to be understood, and responded to upon receipt, compared with emails that are often not viewed by individuals upon receipt [31]. Some participants in the UK RCT reported that text messages about smoking in the intervention arm did stimulate craving [32]. In our study, both arms received smoking cessation advice and could see encouraging messages if they logged on to the website, according to their smoking cessation status.

The anticipated beneficial features of the mobile phone text messages compared with emails did neither result in a different response rate nor a different achieved 7-day self-reported PPA at 6 months post cessation.

### Strengths

The main strengths are that our RCT is nationwide, double-blinded, large, fully automated and conducted close to a real-world setting. We believe that these features are important requirements for any smoking cessation intervention at a population level. We were able to show that a large RCT could be fully automated so that the researchers did not have to interact with the participants.

All our efficacy comparisons are from ITT analyses, and the results should be considered to be conservative measures of the effect of the smoking cessation intervention [23]. We have a high internal validity for comparing the 2 different delivery methods, as the messages in the text message and email arms were identical. We also consider as strengths the computerized randomization and the 2 dispatcher servers for privacy protection.

### Limitations

Our study has several limitations. One limitation is the loss to follow-up and another the low website adherence. However, neither of these differed by arm, making it unlikely that the comparison results are biased. In addition, we use ITT analyses to avoid this bias. The ITT approach reduces the power to detect differences between the 2 arms, therefore increasing the likelihood of not revealing a true difference. Furthermore, we did not utilize the email capabilities of longer and more complex smoking cessation messages as we did not know if this was an advantage or not. It can be argued that this creates an artificial situation that sacrifices external validity for internal validity. It will always be a trade-off between internal (control) and external (allowing real-world applications) validity. In this study, we decided to have the 2 arms as similar as possible and to focus on the delivery methods.

We experienced a technical error during our trial, as they did in the study by Westmaas et al [14]. We consider continuous technical monitoring and support to be crucial, so technical errors can be discovered and fixed when they occur.

During the last part of our trial, smartphones with email functionality that the normal mobile phone did not have, became more common in Norway. This converging of technologies may have blurred the distinction between the emails and text messages during the last part of the study.

### Implications for Future Research

Our study included only Norwegians, of which the majority had more than a high school level of education. Norway has had a good and strict tobacco control policy for many years [33]. Thus, we do not know if our results may be generalized to other racial and ethnic groups, to those with less education, or to those living in countries with no or limited tobacco control policy. In developed countries, most smokers have both a mobile phone and an email account, but this may not be the case in developing countries. Our results are promising, as text messaging is used by most adults in both the developed and developing countries. We encourage the further study of mobile phone–based smoking cessation interventions in low- and middle-income countries.

### Conclusions

This nationwide, double-blinded, large, fully automated RCT found that 1 in 9 enrolled smokers reported 7-day PPA in both arms, 6 months post cessation. Our study found that identical smoking cessation interventions delivered by mobile text messaging and emails may be equally successful at a population level.

## Appendix

Multimedia Appendix 1 CONSORT-EHEALTH checklist (V 1.6.1).

## Abbreviations

eHealth: electronic health
ITT: intention-to-treat
OR: odds ratio
PPA: point prevalence abstinence
RCT: randomized controlled trial
